# Genome-Wide Association Mapping of Salinity Tolerance at the Seedling Stage in a Panel of Vietnamese Landraces Reveals New Valuable QTLs for Salinity Stress Tolerance Breeding in Rice

**DOI:** 10.3390/plants10061088

**Published:** 2021-05-28

**Authors:** Thao Duc Le, Floran Gathignol, Huong Thi Vu, Khanh Le Nguyen, Linh Hien Tran, Hien Thi Thu Vu, Tu Xuan Dinh, Françoise Lazennec, Xuan Hoi Pham, Anne-Aliénor Véry, Pascal Gantet, Giang Thi Hoang

**Affiliations:** 1 National Key Laboratory for Plant Cell Biotechnology, Agricultural Genetics Institute, LMI RICE-2, Hanoi 00000, Vietnam; leducthao@agi.vaas.vn (T.D.L.); pcj.love96@gmail.com (H.T.V.); hienlinh.tran@gmail.com (L.H.T.); xuanhoi.pham@gmail.com (X.H.P.); 2 UMR DIADE, Université de Montpellier, IRD, 34095 Montpellier, France; floran.gathignol@gmail.com (F.G.); francoise.lazennec@umontpellier.fr (F.L.); 3 Faculty of Agricultural Technology, University of Engineering and Technology, Hanoi 00000, Vietnam; nl.khanh@vnu.edu.vn; 4 Department of Genetics and Plant Breeding, Faculty of Agronomy, Vietnam National University of Agriculture, Hanoi 00000, Vietnam; vtthien@vnua.edu.vn; 5 Incubation and Support Center for Technology and Science Enterprises, Hanoi 00000, Vietnam; dinhxt@gmail.com; 6 UMR BPMP, Univ Montpellier, CNRS, INRAE, Institut Agro, 34060 Montpellier, France; anne-alienor.very@cnrs.fr; 7 Department of Molecular Biology, Centre of the Region Haná for Biotechnological and Agricultural Research, Palacký University Olomouc, Šlechtitelů 27, 783 71 Olomouc, Czech Republic

**Keywords:** rice, GWAS, salinity tolerance, Vietnamese landraces, QTL

## Abstract

Rice tolerance to salinity stress involves diverse and complementary mechanisms, such as the regulation of genome expression, activation of specific ion-transport systems to manage excess sodium at the cell or plant level, and anatomical changes that avoid sodium penetration into the inner tissues of the plant. These complementary mechanisms can act synergistically to improve salinity tolerance in the plant, which is then interesting in breeding programs to pyramidize complementary QTLs (quantitative trait loci), to improve salinity stress tolerance of the plant at different developmental stages and in different environments. This approach presupposes the identification of salinity tolerance QTLs associated with different mechanisms involved in salinity tolerance, which requires the greatest possible genetic diversity to be explored. To contribute to this goal, we screened an original panel of 179 Vietnamese rice landraces genotyped with 21,623 SNP markers for salinity stress tolerance under 100 mM NaCl treatment, at the seedling stage, with the aim of identifying new QTLs involved in the salinity stress tolerance via a genome-wide association study (GWAS). Nine salinity tolerance-related traits, including the salt injury score, chlorophyll and water content, and K^+^ and Na^+^ contents were measured in leaves. GWAS analysis allowed the identification of 26 QTLs. Interestingly, ten of them were associated with several different traits, which indicates that these QTLs act pleiotropically to control the different levels of plant responses to salinity stress. Twenty-one identified QTLs colocalized with known QTLs. Several genes within these QTLs have functions related to salinity stress tolerance and are mainly involved in gene regulation, signal transduction or hormone signaling. Our study provides promising QTLs for breeding programs to enhance salinity tolerance and identifies candidate genes that should be further functionally studied to better understand salinity tolerance mechanisms in rice.

## 1. Introduction

More than one third of cultivated lands are polluted by excess of salt (NaCl) [1]. Sodium is a toxic element for plants and this is particularly true for rice, which is often cultivated in river delta areas where irrigation water is increasingly frequently contaminated by sea water [2]. Rice is the most important food crop, feeding more than three billion people in the world [3]. In Vietnam, rice occupies 85% of the total agricultural area [4]. However, with 3620 km of coastline spreading from north to south, Vietnam has been ranked among the top five countries likely to be most affected by climate change [5]. Vietnam is one of the first rice exporters in the world and consequently plays an important role in food supply security, particularly in Asian countries [6,7]. The Mekong River Delta and Red River Delta are the main areas of rice production in Vietnam; the Mekong River Delta represents 50% of the total rice production area and supplies 90% of the rice exported by the country [7]. The Mekong River Delta is increasingly menaced by an elevation in salinity due to sea water intrusion that results from different climatic and anthropic factors [8]. According to data from the Ministry of Science and Technology of Vietnam, at the end of 2015 and the first months of 2016, saline intrusion in the Mekong River Delta reached the highest level measured during the past 100 years. In addition to global management of the causes leading to increases in salinity, it is important to breed new varieties of rice tolerant to salinity, which necessitates the identification of genetic determinants conferring salinity tolerance [9]. Several salinity tolerance QTLs (quantitative trait loci) have been identified in rice using association genetics approaches, and the mechanisms undelaying rice salinity tolerance start to be well known (for reviews see [10,11]). The mechanisms involved in rice salinity tolerance act at different levels and combine transcriptional and posttranscriptional or posttranslational regulation events that lead to sodium exclusion or compartmentation in specific cell infrastructures, osmolyte production or anatomical changes that avoid sodium penetration into the internal tissues of the plant [10]. These mechanisms act in different complementary ways that synergistically allow salinity tolerance [10]. For these reasons, it is interesting to combine genetic sources with different and complementary salinity tolerance to increase resistance to salinity, which can also buffer the susceptibility of QTL effects to environmental conditions [12,13]. To identify such complementary sources of salinity tolerance, it is necessary to look for them in the widest and most diverse panels of varieties possible.

To contribute to this goal, in this study, we screened a genotyped panel of 179 Vietnamese landrace varieties of indica and japonica rice collected in different agrosystems from North to South Vietnam for salinity tolerance [14]. Vietnamese landrace varieties are often underrepresented in the studied international panels such as the 3K panel developed by the International Rice Research Institute (IRRI), even though, they potentially constitute an original source of valuable alleles [15,16]. We already used this panel to identify valuable QTLs associated with root, leaf or panicle traits and water deficit tolerance by genome wide association study (GWAS) [17,18,19,20]. The plants were screened for salinity stress tolerance at an early developmental stage using a hydroponic culture system in the presence of 100 mM NaCl. The phenotypic standard evaluation score (SES) [21], chlorophyll and relative water content and the concentrations of K^+^ and Na^+^ ions were measured in leaves. GWAS revealed 26 QTLs including 10 QTLs associated with several traits. Most of these QTLs contain candidate genes that may explain their effect on salinity tolerance, and the function of the genes are further discussed.

## 2. Results

### 2.1. Phenotypic Variation and Heritability of Salinity Tolerance-Related Traits

The phenotyping experiment was conducted for three consecutive years, from 2017 to 2019. The observed salt tolerance diversity in different accessions was reproducible. The data from the last trial were chosen for performing GWAS, for which the screening protocol was improved and standardized for the Vietnamese rice landrace panel and for the parameter measurement as described in the Materials and Methods section. In this trial, on the tenth day after the start of salinization, 25 plots of 19 accessions were monitored to have simultaneously reached a score of 7, which included the susceptible check IR29. A total of 9 salinity tolerance-related traits were evaluated, three of which (leaf water content (WC), chlorophyll a to chlorophyll b ratio in leaves (Chla_b), and ratio of Na^+^/K^+^ in leaves (Na_K)) were computed from the directly measured traits. Statistical analysis was conducted for the full panel and the indica and japonica subpanels (Table 1). Within the full panel and the indica subpanel, significant replication and genotypic effects were observed for most of the traits, with the exception of Chla_b. Meanwhile, the genotypic effect for the chlorophyll traits of the japonica subpanel was insignificant (Table 1). The broad-sense heritability (H^2^) calculated for each trait with a significant genotypic effect was moderate to high, varying from 0.40 to 0.76, while high values were recorded for WC, Score and three ion content traits.

**Figure 1:**
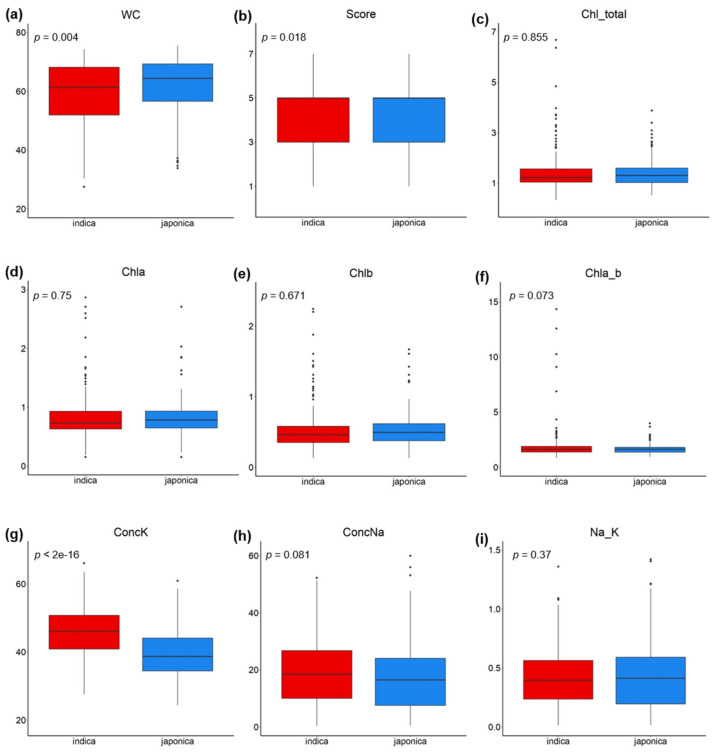
Boxplots of the distribution of salinity tolerance-related traits. Indica subpanel in red; japonica subpanel in blue; statistical significance (ANOVA *p*-values) between the two subpanels is indicated; (**a**) leaf water content (WC); (**b**) score of visual salt injury (Score); (**c**) total chlorophyll content in leaves (Chl_total); (**d**) chlorophyll a content in leaves (Chla); (**e**) chlorophyll b content in leaves (Chlb); (**f**) chlorophyll a to chlorophyll b ratio in leaves (Chla_b); (**g**) K^+^ concentration in leaves (ConcK); (**h**) Na^+^ concentration in leaves (ConcNa); (**i**) ratio of Na^+^/K^+^ in leaves (Na_K).

Significant phenotypic variation was observed for all of the traits, with “full name” (CVs) ranging from 15.35% to 70.47% (Table 1). Figure 1 shows statistically significant differences in the mean values of WC, Score and ConcK between the indica and japonica subpanels. Specifically, the indica subpanel displayed a lower WC and higher Score and ConcK than the japonica subpanel (Table 1, Figure 1). Consequently, for the Vietnamese rice landrace panel used in this study, indica accessions were considered less salt-tolerant than japonica accessions.

The correlations among the traits determined the same tendency within the full panel and the two subpanels (Figure S1). However, the correlation coefficients were largely variable between the traits (Table 2). For instance, Score, ConcNa and Na_K were strongly negatively correlated with WC. ConcNa and Na_K were also highly correlated with Score. In contrast, ConcK constituted weak correlations with the other traits, except for a moderate correlation with WC. Overall, higher correlations were observed among WC, Score, ConcNa and Na_K.

### 2.2. SNP-Trait Associations

**Figure 2:**
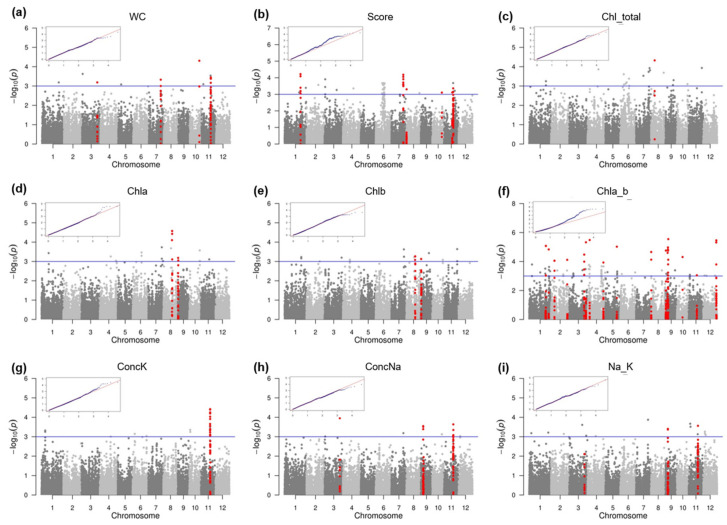
Manhattan plots and Q-Q plots for GWAS of salinity tolerance-related traits in the full panel. (**a**) Leaf water content (WC); (**b**) score of visual salt injury (Score); (**c**) total chlorophyll content in leaves (Chl_total); (**d**) chlorophyll a content in leaves (Chla); (**e**) chlorophyll b content in leaves (Chlb); (**f**) chlorophyll a to chlorophyll b ratio in leaves (Chla_b); (**g**) K^+^ concentration in leaves (ConcK); (**h**) Na^+^ concentration in leaves (ConcNa); (**i**) ratio of Na^+^/K^+^ in leaves (Na_K). In the Manhattan plots, significant SNPs are highlighted in red.

GWAS analyses were conducted for the full panel and for the indica and japonica subpanels separately. The GWAS results are presented in the Q-Q and Manhattan plots in Figure 2 and Figure S2. Using the *p*-value threshold of 1 × 10^−4^, we identified 64 associations between 58 SNPs and the studied traits, but no associations were detected in the japonica subpanel. These 58 significant SNPs were distributed in 26 QTL regions. Within the detected QTL regions, the number of significant SNPs increased to 119 when the threshold value was set at 1 × 10^−3^ (Table S1). Among these values, 110 SNPs were found in the full panel, 44 were identified in the indica subpanel, and 35 were common between the full panel and the indica subpanel.

A total of 16 QTLs were associated with Chla_b, 6 with WC, 6 with Score, 4 with ConcNa, 3 with Chl_total, 3 with Chlb, 2 with Chla, 3 with Na_K, and 1 with ConcK (Table 3). Ten of the 26 identified QTLs were associated with multiple traits, including QTL_25 on chromosome 11 associated with five traits (i.e., WC, Score, ConcK, ConcNa, and Na_K); QTL_21 on chromosome 9 associated with 4 traits (i.e., WC, Chla_b, ConcNa, and Na_K); three QTLs (QTL_9, QTL_20, and QTL_23) associated with three traits; and five other QTLs (i.e., QTL_13, QTL_16, QTL_17, QTL_19, and QTL_24) associated with two traits. Most of the individual trait-associated QTLs were detected for chlorophyll traits, except for QTL_1, which was related to Score. The number of significant SNPs within each QTL varied from 1 to 33, whereas QTL_25 was defined by 33 SNPs, QTL_21 by 14 SNPs, QTL_1 and QTL_4 by 8 SNPs, and QTL_16 and QTL_19 by 7 SNPs (Table 3).

**Figure 3:**
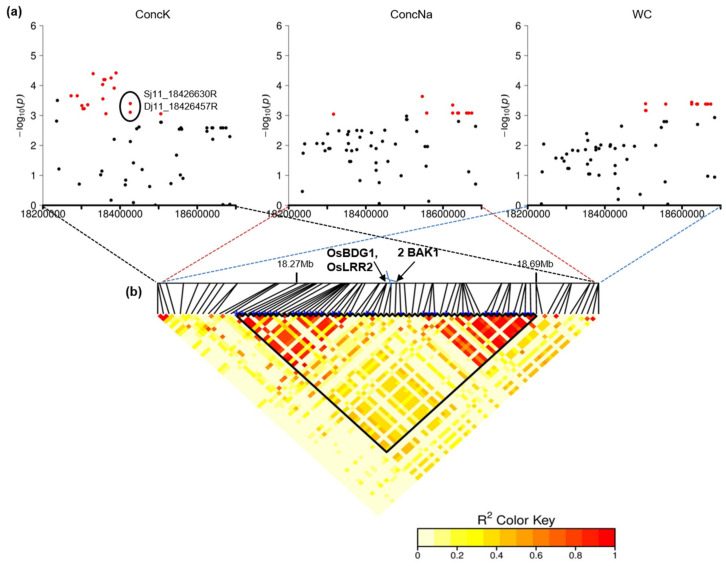
QTL_25. (**a**) Manhattan plot for K^+^, Na^+^ and water content in leaves in the full panel; (**b**) linkage disequilibrium (LD) heatmap. In the Manhattan plots, significant SNPs are highlighted in red, and genes of interest are mentioned. The genomic region of QTL_25 is specified in the boundary area in the LD heatmap.

Therefore, among the 26 detected QTLs, QTL_25 was supposed to be the major QTL due to being mapped by the highest number (33) of significant SNPs and associated with the greatest number (5) of traits in both the full panel and the indica subpanel (Figure 3). The next was QTL_21, which was common to 4 traits and supported by 14 significant SNPs.

### 2.3. Colocalizing QTLs and Candidate Genes Underlying the Detected QTLs Involved in Salinity Tolerance

The sites of the QTLs identified in this study were compared with QTLs detected in mapping populations and derived by other GWASs related to salinity tolerance. Consequently, most of our QTLs colocalized with already known QTLs, except for QTL_6, QTL_17 and QTL_22 (Table S2). We found a total of 100 colocalizations, of which 17 were detected by GWAS [22,23,24,25,26], and 83 other colocalizations were mapped in biparental populations [13,27,28,29,30,31,32,33,34,35,36,37,38,39,40,41,42,43,44,45]. In particular, 8 colocalizations shared similar traits (leaf chlorophyll content, K^+^ concentration, Na^+^ concentration, leaf water content). In addition, colocalizations with QTLs identified in previous studies for other traits using the same Vietnamese rice panel and genotyping data were observed (Table S3). For the latter, forty-nine overlapping associations were found that underlie QTL_3, QTL_6, QTL_8, QTL_17, QTL_20, and QTL_26.

In the region of almost all QTLs identified, a number of candidate genes related to the response of plants to salt or abiotic stress were found, with the exception of QTL_7, QTL_11, QTL_14, QTL_15, QTL-17, QTL_18 and QTL_22 (Table 3). These candidate genes encode different kinds of proteins including transcription factors, receptor-like kinases (RLKs), mitogen-activated kinase (MAPK), enzymes and transporters.

**Table 3:** List of candidate genes located within the identified QTLs.

QTL Name	Chr	QTL Position (bp)	Panel	Traits	No. of Signif. SNPs	Candidate Gene				References
						Locus	Gene	Protein	Description	
QTL_1	1	31,557,933–31,695,659	F	Score	8	*Os01g54890*	*OsERF922*	Ethylene-responsive transcription factor 2	Ethylene response transcription factor, negative regulation of salt resistance	[46]
						*Os01g54930*	*OsVOZ1/EIP8*		Vascular one zinc-finger 1/EBR1-interacting protein 8	
QTL_2	1	32,165,198–33,076,887	F	Chla_b	6	*Os01g55940*	*OsGH3.2*	IAA-amido synthetase	Modulation of free IAA and ABA homeostasis and drought and cold tolerance	[47]
						*Os01g55974*	*STRIPE 2, ALR*	Deoxycytidylate deaminase	Chloroplast development	[48,49]
						*Os01g56040*	*OsSAP3*	A20/AN1 zinc-finger protein 3	Inducibility to drought and salinity stress	[50]
						*Os01g56070*	*OsRDCP3*	RING finger protein 5	increase tolerance to drought stress in rice	[51]
						*Os01g56400*	*OsABCI6*	ABC transporter ATP-binding protein	response to abiotic stress	[52,53]
						*Os01g56680*	*PsbW*	Photosystem II reaction center W protein	photosynthesis regulation	[54]
QTL_3	1	38,515,041–38,722,651	F, I	Chla_b	1	*Os01g66590*	*OsAS2*	LOB domain protein	Regulation of shoot differentiation and leaf development	[55]
						*Os01g66610*		Lectin receptor-like kinase (LecRLK)	Regulation of plant growth and developmental processes in response to stress	[56]
						*Os01g66420*	*OsPHD7*	PHD finger protein (ZF-TF)	Up-regulated under drought stress	[57]
QTL_4	2	6,668,466–6,853,020	F, I	Chla_b	8	*Os02g12750*	*OsTET2*	Tetraspanin domain containing protein	Response to heat, salt and water deficit stresses at seedling stage	[58]
						*Os02g12790*	*OsCga1*	GATA transcription factor (ZF-TF)	Regulation of chloroplast development and plant architecture, relating to natural variation in strong stay-green	[59,60]
						*Os02g12794*	*eEF-1B gamma*	Elongation factor 1-gamma	Salinity stress adaptation	[61]
						*Os02g12800*	*EF-1gamma*	Elongation factor 1-gamma	Salinity stress adaptation	[61]
QTL_5	2	32,011,340–32,679,510	F	Chla_b	1	*Os02g52290*	*OsFKBP12*	Peptidyl-prolyl cis-trans isomerase	Salinity stress response	[62,63]
						*Os02g52650*	*LhCa5*	Chlorophyll a/b-binding protein	Light-harvesting chlorophyll a/b-binding protein	[64]
						*Os02g52670*	*OsERF#103*	Ethylene-responsive transcription factor	Responsive to drought and salinity stress	[65]
						*Os02g52744*		DCL chloroplast precursor		
						*Os02g52780*	*OsbZIP23*	bZIP transcription factor	Regulation of ABA signaling and biosynthesis, salinity and drought tolerance	[66,67]
						*Os02g53030*	*OsRLCK84*	MAPK kinase	Response to salinity stress	[68]
QTL_6	3	526,748–1,177,466	I	Chla_b	1	*Os03g02010*	*OsDRM2*	DNA methyltransferase	Tissue- and genotype-dependent response to salinity stress	[69]
						*Os03g02280*	*OsS40-4*	S40-like protein	Response to leaf senescence and salinity stress	[70,71]
						*Os03g02590*	*OsPEX11-1*	Peroxisomal biogenesis factor 11	Relating to leaf senescence, salt responsive	[72,73]
QTL_7	3	7,197,414–7,297,414	I	Chlb	1					
QTL_8	3	29,719,291–29,898,084	F, I	Chla_b	5	*Os03g51970*	*OsGRF6*	Growth-regulating factor	Targeted by osa-miR396 and drought-up sRNA56202 responsive to salt and drought stress	[74,75,76]
						*Os03g52090*	*OsECA2*	Calcium-transporting ATPase 3	P-type Ca2+ ATPase IIA, harboring multiple stress-induced cis-acting elements	[77]
QTL_9	3	30,313,283–30,481,199	F	WC, ConcNa, Na_K	1	*Os03g53060*				
QTL_10	3	33,128,341–33,501,467	F	Chla_b	1	*Os03g58250*	*OsbZIP33*	bZIP transcription factor	ABA-dependent enhancer of drought tolerance, responsive to high salinity, H2O2 and high temperature stress	[78]
						*Os03g58300*	*OsIGL*	Indole-3-glycerol phosphate lyase	Chloroplast precursor	
						*Os03g58390*	*OsSIRP2*	RING Ub E3 ligase	Salt and osmotic stress tolerance enhancer	[79]
						*Os03g58540*	*TSV3/OsObgC2*	Obg-like GTPase protein	Chloroplast development at the early leaf stage under cold stress	[80]
QTL_11	4	4,254,414–4,354,414	F, I	Chla_b	1					
QTL_12	4	31,433,085–31,558,275	F, I	Chla_b	1	*Os04g52960*	*OsNUC1*	Nucleolin-like protein	Photosynthesis adaptation, reduction of oxidative stress and yield loss under salinity stress, enhancement of salt-stress tolerance	[81,82]
QTL_13	5	22,437,918–22,840,944	F, I	WC, Chla_b	2	*Os05g38370*	*OsFKBP20-1a*	Peptidyl-prolyl cis-trans isomerase FKBP-type	Drought and heat stress-response	[83]
						*Os05g38290*	*OsPP2C49*	Protein phosphatase 2C	Regulation of ABA-mediated signaling pathways	[84]
QTL_14	7	7,040,925–7,140,925	I	Chl_total	1					
QTL_15	7	21,360,003–21,460,003	I	Chl_total	1					
QTL_16	7	23,502,762–23,623,244	F	WC, Score	7	*Os07g39270*	*OsGGPPS1*	Geranylgeranyl pyrophosphate synthase	Chlorophyll biosynthesis	[85]
						*Os07g39350*		Sugar transporter	osmo protection	
						*Os07g39360*		Sugar transporter	osmo protection	
QTL_17	8	235,171–472,039	F, I	Score, Chla_b	4					
QTL_18	8	7,116,026–7,249,222	F	Chl_total	1					
QTL_19	8	17,191,665–17,648,853	F, I	Chla, Chlb	7	*Os08g28710*	*OsRLCK253*	Receptor-like kinase	Improvement of water-deficit and salinity stress tolerance	[86]
QTL_20	9	799,160–1,286,768	F	Chla, Chlb, Chla_b	5	*Os09g02270*	*OsCYL4*	Protein containing cyclase domain	Negative regulation of abiotic stress tolerance in relation to accumulation of ROS	[76]
QTL_21	9	4,452,802–5,809,538	F, I	WC, Chla_b, ConcNa, Na_K	14	*Os09g10600*		NADH-dependent enoyl-ACP reductase	Chloroplast precursor	
QTL_22	10	11,126,654–11,242,896	F	Chla_b	1					
QTL_23	10	18,944,166–19,070,983	F	WC, Score, ConcNa	2	*Os10g35640*		Rf1 mitochondrial precursor (Nin-like)	Down-regulated salt-responsive, up-regulated cold-responsive	[87,88]
						*Os10g35560*	*OsSFR6*	Expressed protein	Osmotic stress and chilling tolerance	[89]
QTL_24	11	16,335,298–16,441,782	F, I	Score, Chla_b	1					
QTL_25	11	18,273,105–18,684,503	F, I	WC, Score, ConcK, ConcNa, Na_K	33	*Os11g31530*	*OsBDG1*	BRASSINOSTEROID INSENSITIVE 1-associated receptor kinase 1, OsBri1	Salinity tolerance (upregulated in roots in response to salinity)	[90]
						*Os11g31540*	*OsLRR2*	BRASSINOSTEROID INSENSITIVE 1-associated receptor kinase 1, OsBri1	Stress tolerance (upregulated in leaves in response to cold and drought stress)	[91]
						*Os11g31550*		BRASSINOSTEROID INSENSITIVE 1-associated receptor kinase 1, OsBri1		
						*Os11g31560*		BRASSINOSTEROID INSENSITIVE 1-associated receptor kinase 1, OsBri1		
QTL_26	12	25,841,227–26,215,713	F	Chla_b	5	*Os12g41860*	*OsHox33*	HDZIP III transcription factor	Targeted by a miRNA responsive to salinity stress, control of leaf senescence	[75,92]
						*Os12g41950*	*OsARF6b, OsARF25*	Auxin response factor	Candidate salinity tolerance-related gene at the seedling stage	[93]
						*Os12g42060*	*OsWAK128*	OsWAK receptor-like kinase	Candidate salinity tolerance-related gene at the seedling stage	[93]
						*Os12g42070*	*OsRLCK375, OsWAK129*	OsWAK receptor-like kinase	Down-regulated in cold, salt and drought stress conditions at the seedling stage	[94]
						*Os12g42090*		37 kDa inner envelope membrane protein	Chloroplast precursor, salinity-inducible	[93,95]
						*Os12g42200*	*OsCHX15*	ATCHX protein	Cation H+ antiporter, candidate salinity tolerance-related gene at the seedling stage	[93]
						*Os12g42250*	*OsZFP213, PINE1*	C2H2 transcription factor	Interacting with OsMAPK3 to enhance salinity tolerance by enhancing ROS-scavenging ability, regulating internode elongation and photoperiodic signals	[96,97]

## 3. Discussion

Rice is considered to be very sensitive to salinity [98,99]. Here, to determine the response of the Vietnamese rice landrace panel to salinity, a moderate salinity stress (100 mM NaCl) was applied at the seedling stage. We assessed a total of 9 phenotypic traits, all of which showed high variability within the panel in response to salinity stress. Of these 9 traits, WC, Score and three ion content traits (ConcNa, ConcK and Na_K) exhibited high heritability (0.60–0.76). Additionally, strong correlations (0.59–0.97) were observed among these traits with the exception of ConcK, indicating that WC, Score, ConcNa and Na_K were strongly associated with the response of rice plants to salinity stress, which is consistent with previous studies on rice salinity tolerance evaluation [100,101,102]. WC is a physiological parameter of the plant water status that expresses the response of plants to osmotic stress [103,104], ionic content traits reflect the level of ionic stress (ion homeostasis) [105], and the salt injury score is an indicator of plant damage/survival (growth performance) under salinity stress [100]. Previous studies reported that rice accessions tolerant to salinity stress have the ability to reduce the osmotic stress, prevent the excess accumulation of Na^+^ and absorb greater K^+^ to maintain a low shoot Na^+^/K^+^ ratio [106,107,108,109].

Correlations among the traits varied in the same direction in the full panel and the two subpanels. However, the japonica subpanel had, on average, greater WC and lower Score than the indica subpanel, indicating that japonica accessions are more salt-tolerant than indica accessions. This finding contradicts the results reported in a previous study [101] that used 4 japonica varieties and 6 indica varieties. This contradiction can be explained by the difference in the number of rice accessions included in the screening. In our study, 112 indica and 64 japonica accessions were evaluated.

In this study, GWAS analyses were applied for the full panel and for the two subpanels. Thus far, we succeeded in identifying 119 significant SNPs assigned to 26 QTLs. Twenty-two QTLs were detected in the full panel and 15 QTLs were detected only in the indica subpanel, but no japonica-specific QTLs were found, although japonica seems to have an average higher salinity tolerance than indica accessions. Similarly, in other studies using the same rice panel and genotyping data screened for water deficit tolerance and leaf traits, no japonica-specific QTLs were detected [17,18], likely because japonica accessions represent only one-third of the total accessions of the panel. In this study, of the 26 identified QTLs, 11 QTLs were detected in both the full panel and the indica subpanel, and 10 QTLs were associated with two or more traits (Table 3). Interestingly, all the QTLs that were detected for WC colocalized with QTLs associated with Score and/or ion content traits, except for QTL_13, which was found for WC and Chla_b. These results suggest that WC, Score and ion content traits have a shared genetic basis related to salinity stress responses. However, there was no overlap between QTLs detected for chlorophyll-related traits and ion content traits.

The QTLs discovered in this study were located on most chromosomes, apart from chromosome 6, and we found 3 QTLs (QTL_1, QTL_2 and QTL3) on chromosome 1, but none of these QTLs colocalized with *Saltol*, a well-known major QTL for rice salinity tolerance at the seedling stage [110,111]. A large number of QTLs for salinity tolerance detected in this study colocalized with QTLs detected in other studies and populations under conditions of salt stress at vegetative or reproductive stages, which validates our approach (Tables S2 and S3). Interestingly, of 26 QTLs identified in the present study, 3 QTLs (QTL_6, QTL_17 and QTL_22) did not colocalize with previously reported QTLs and thus constituted novel QTLs. They can be of high interest to bring new salinity tolerance sinks into breeding programs.

The major QTL identified in our study, QTL_25 at 18,273.1–18,684.5 kb on chromosome 11 was associated with WC, Score, ConcK, ConcNa and Na_K (Table 3, Figure 3). In particular, QTL_25 was mapped by 33 significant SNPs, and each of them contributed 5.75–14.09% to the phenotypic variation (Table S1). QTL_25 colocalized with previously identified QTLs under conditions of salinity stress using different mapping populations (Table S2), i.e., with 2 QTLs associated with leaf water content [42], with QTL qSHL11.1 for shoot length and QTL qRTL11.1 for root length [39], and with 4 GWAS-derived QTLs for the number of unfilled grains per plant [25] (Table S2), suggesting that this QTL has a pleiotropic effect on plant growth and reproduction under salinity stress and likely acts synergistically with other major salinity tolerance QTLs such as *Saltol*, in enhancing the salinity tolerance in rice.

Compared to the previous GWAS [14,17,18,20,112], using the same rice panel and genotyping data, we found 28 associations of 6 QTLs identified in this study colocalized with 23 associations of [14,17,20,112], but there was no colocalization between QTLs for salinity tolerance-related traits and for leaf mass traits [18] (Table S3). Remarkably, 9 associations for various drought tolerance-related traits, including relative water content after 2 and 3 weeks of drought stress, slope of relative water content after 2 weeks of drought stress, drought sensitivity score after 2, 3 and 4 weeks of drought stress, and recovery ability, belonging to QTL q9 of [17], were colocalized with all associations in QTL_17 for Chla_b and Score, suggesting that this genomic region contains important genetic determinants for rice adaptation to osmotic stresses.

Underlying 19 out of the 26 QTLs detected in this study, a high number of genes were annotated or functionally associated with salinity tolerance (Table 3).

Most candidate genes encode transcription factors reported to be involved in the rice response to salinity or abiotic stresses. Found in QTL_1 for Score, the *OsERF922* gene (*ETHYLENE RESPONSE FACTOR 922*, *Os01g54890*) negatively regulates tolerance to salinity stress through an ABA signaling pathway, since rice transgenic plants overexpressing *OsERF922* exhibited reduced salinity tolerance with increased shoot Na^+^/K^+^ ratio and ABA level, and knockdown of *OsERF922* expression reduced the ABA accumulation [46]. Additionally, as a member of the ERF gene family, *OsERF922*, the expression of *OsERF#103* (*ETHYLENE RESPONSE FACTOR 103*, *Os02g52670*) (in QTL_5), was reported to be upregulated under drought and salinity stress conditions at the seedling stage [65].

Furthermore, we found two potential genes encoding bZIP transcription factors. In plants, bZIP genes are involved in the response to abiotic stress [66,113]. One of these two genes is *OsbZIP23* (*b-ZIP TRANSCRIPTION FACTOR 23, Os02g52780*) (in QTL_5), which was functionally characterized as being an ABA-dependent enhancer of drought and salinity tolerance [66,67]. On the one hand, *OsbZIP23* overexpression significantly enhances tolerance to drought stress, especially to high salinity stress, compared with the wild type [66,67]. On the other hand, the *OsbZIP23* mutant displays significantly reduced tolerance to drought and salinity stress [67]. In addition, the SUMO protease *OsOSTS1* (*OVERLY TOLERANT TO SALT 1*), a gene involved in tolerance to high salinity [114], was reported to directly target OsbZIP23, which results in activation of OsbZIP23 and stimulation of OsbZIP23-dependent gene expression, which helps promote tolerance to drought stress [115]. Similar to *OsbZIP23*, *OsbZIP33* (*b-ZIP TRANSCRIPTION FACTOR 33*, *Os03g58250*), located in QTL_10, also plays a role as an ABA-dependent enhancer of drought and salinity tolerance. *OsbZIP33* is highly upregulated under drought and high salinity stress conditions. *OsbZIP33*-overexpressing transgenic plants exhibited significantly increased drought tolerance [78].

Three candidate genes belonging to the zinc-finger transcription factors were identified: *OsSAP3* (*STRESS-ASSOCIATED PROTEIN 3*, *Os01g56040*) in QTL_2, *OsPHD7* (*PHD FINGER PROTEIN 7*, *Os01g66420*) in QTL_3, and *OsCga1* (*CYTOKININ GATA TRANSCRIPTION FACTOR 1*, *Os02g12790*) in QTL_4. *OsSAP3* and *OsPHD7* are related to abiotic stress responses. In particular, the expression of *OsSAP3* is induced in response to drought and salinity stress [50], and *OsPHD7* is upregulated under drought stress [57]; moreover, *OsCga1* is associated with the development of chloroplasts [59] and stay-green [60]. Stay-green refers to the ability to maintain green leaves and photosynthetic capacity and is thus related to plant adaptation to osmotic stress [116]. Overexpression of *OsCga1* delays leaf senescence [59].

Underlying QTL_2, *OsRDCP3 (RING DOMAIN-CONTAINING PROTEIN 3*, *Os01g56070*) was predicted to be involved in drought stress tolerance [51], and *OsABCI6* (*ABC TRANSPORTER I FAMILY MEMBER 6*, *Os01g56400*) was supposed to be involved in the response to abiotic stress [52,53]. Similarly, the expression of *OsTET2* (*TETRASPANIN 2*, *Os02g12750*), an integral membrane protein found in QTL_4, was increased in drought-stress seedlings; in addition, this gene was highly upregulated under heat and salinity stress [58].

Two other candidate transcription factor genes were found in QTL_26 on chromosome 11, including *OsHox33* (*HOMEOBOX GENE 33*, *Os12g41860*) and *OsARF25* (*AUXIN RESPONSE FACTOR 25*, *Os12g41950*). *OsHox33*, encoding an HDZIP transcription factor, is involved in leaf senescence because its knockdown accelerates leaf senescence [92] and is a target of a salinity stress-responsive miRNA [75]. *OsARF25* is also a salinity tolerance-related candidate gene discovered by GWAS, as reported by [93].

Another transcription factor gene identified, *OsAS2* (*ASYMMETRIC LEAVES 2*, *Os01g66590*) in QTL_3 [55], was associated with the development of plants. *LhCa5* (*PHOTOSYSTEM I LIGHT HARVESTING COMPLEX GENE 5*, *Os02g52650*) in QTL_5 was predicted to function in the photosystem [64].

Within the region of QTL_25, the strongest QTL found in this study, we detected a consecutive set of four *BRASSINOSTEROID INSENSITIVE 1-associated receptor kinase 1 (BAK1)*, including *Os11g31530* (*OsBDG1*), *Os11g31540* (*OsLRR2*), *Os11g31550*, and *Os11g31560* (Figure 3b). *BAK1*, encoding a leucine-rich repeat type II receptor-like kinase, functions as a coreceptor of BRI1 in brassinosteroid plant signaling [117]. Perception of brassinosteroids through the BRI1-BAK1 complex can influence the growth and development of rice plants [118], e.g., regulating the leaf angle and grain size [119] and regulating ABA-induced stomatal closure, which is critical for the survival of plants under water stress [120]. Among these four *BAK1* genes, *OsBDG1* and *OsLRR2* are considered to be involved in salt and/or abiotic stress responses [90,91]. Under salinity stress conditions, *OsBDG1* is significantly upregulated in roots of the rice-sensitive cultivar IR29, whereas *OsLRR2* is upregulated in roots of the rice-tolerant cultivar FL478 [90]. Additionally, the expression of *OsLRR2* is highly induced in leaves after cold and drought treatment; thus, *OsLRR2* is a supposed candidate gene involved in tolerance to abiotic stress [91]. Interestingly, two significant SNPs identified in this study, Sj11_18426630R and Dj11_18426457R, were located in the sequence of *OsBDG1* (Figure 3). Dj11_18426457R is intronic, while Sj11_18426630R is positioned within a coding sequence (i.e., exon 5) that changes the amino acid sequence in the LRR domain of the OsBDG1 protein. Thus, the perspective of a functional characterization of these *BAK1* candidate genes is opened.

Three other genes encoding receptor-like kinase (RLK) with enhanced abiotic stress tolerance are *Os08g28710* (*OsRLCK253*) in QTL_19 and *Os12g42060* (*OsWAK128*) and *Os12g42070* (*OsRLCK375*, *OsWAK129*) in QTL_26. Functionally, *OsRLCK253* confers tolerance to salt and water deficits in transgenic *Arabidopsis thaliana* plants during different growth stages, resulting in yield protection against stress [86]. *OsWAK128* and *Os12g42070* were candidate genes near a GWAS-derived QTL related to salinity tolerance at the seedling stage [93]. In addition, a mitogen-activated protein kinase (MAPK) encoded by the *OsRLCK84* gene (*Os02g53030*) in QTL_5 was activated in response to salinity stress [68].

## 4. Materials and Methods

### 4.1. Plant Materials and Genotyping

This study included 179 Vietnamese rice landraces and 3 control genotypes (Nipponbare, Azucena and IR64). The Vietnamese rice accessions came from diverse locations throughout Vietnam and were originally provided by the Plant Resource Center (21°00′05″N and 105°43′33″E). All 182 accessions were genotyped by 21,623 single nucleotide polymorphism (SNP) markers using genotyping-by-sequencing with a minor allele frequency above 5% [14]. IR29 was used as a susceptibility check for phenotyping experiments. The names of the accessions, provinces of origin and ecosystem are described in Table S4. More detailed information on this panel can be found in [14].

### 4.2. Phenotyping Experiment

#### 4.2.1. Salt Treatment

The experiment was conducted from August 26, 2019, to September 24, 2019, at the Agriculture Genetics Institute, Hanoi, Vietnam (21°02′55″ N and 105°46′58″ E). The accessions were grown in hydroponics following the IRRI standard protocol with three replicates [100]. Within each replicate, the accessions were randomly distributed in 5.2 individual plastic trays (36 × 31 × 15 cm) fitted with styrofoam float of 35 slots (2 mm diameter) filled with Peters solution composed of 1 g/L Peters water-soluble fertilizer (20-20-20 NPK) and 200 mg/L ferrous sulfate [21]. A total of 16 plastic trays were used.

The experiment was set under greenhouse conditions. After breaking dormancy at 50 °C for five days, seeds were soaked in water for 2–3 days. When germination began, seeds were incubated in a culture room (28 °C, photoperiod 12 h light/12 h dark) for 2 days. Once the primary root emerged well at a length of 2–3 cm, seedlings were cultured in styrofoam floats with a nylon net bottom according to the experimental design. Four seedlings were cultured per slot. Three days after seeding, seedlings were thinned to keep 3 well-developed plants per slot. The pH (5.2) and the level of nutrient solution were adjusted daily. The Peters solution was replaced weekly until the end of the experiment. Salinity stress was applied when plants reached the fourth leaf stage. Salt NaCl was gradually supplemented to the hydroponic medium to avoid osmotic shocks. Each time, 50 mM NaCl was separated by two days to obtain a final concentration of 100 mM NaCl. The experiment was stopped once all the plants exhibited drying in most leaves (average evaluation score of 7).

#### 4.2.2. Scoring and Sampling

For each plant, salinity tolerance score was evaluated based on leaf injury symptoms using the modified standard evaluation score (SES) for rice [21], as follows: score 1—normal growth, no leaf symptoms; score 3—near normal growth, but leaf tips or few leaves whitish and rolled; score 5—growth severely retarded, most leaves rolled, only a few elongating; score 7—complete cessation of growth, most leaves dry, some plants dying; score 9—almost all plants dead or dying.

After scoring, the second fully expanded leaves of three plants in each hole were harvested. Quickly cut a 1.5 cm fragment from the leaf base, separately pack the material of each hole in aluminum foil, avoiding folding the leaves, and place on ice for chlorophyll determination. The rest of the cut leaves were immediately put into a small zip plastic bag of known weight for measuring the water content.

#### 4.2.3. Chlorophyll Determination

The chlorophyll content was estimated as described in the protocol of [121] with some modifications. The harvested samples were weighed, put into 2-mL Eppendorf tubes, and ground in liquid nitrogen. The pellet was resuspended in 1.5 mL of 85% acetone solution and centrifuged at 12,000× *g* at 4 °C for 15 min. One milliliter of the supernatant was collected, and the absorbance was measured at wavelengths of 645 and 663 nm using a 7305 UV/visible spectrophotometer (Jenway, Staffordshire, UK). The chlorophyll content was calculated as follows: total chlorophyll (Chl_total, µg/mL) = 20.2 (A_645_) + 8.02 (A_663_), chlorophyll a (Chla, µg/mL) = 12.7 (A_663_) − 2.69 (A_645_), chlorophyll b (Chlb, µg/mL) = 22.9 (A_645_) − 4.68 (A_663_). The values were then converted to the amount of chlorophyll per milligram of fresh tissue (µg/mg). The ratio of chlorophyll a to chlorophyll b (Chla_b) was also determined.

#### 4.2.4. Water Content Measurement

The bags containing samples were weighed to determine the sample fresh weight (FW). After being dried for 3 days at 70 °C in an oven, the sample dry weight (DW) was measured. The leaf water content of each sampling was calculated using the formula: WC (%) = (FW − DW) × 100/FW.

#### 4.2.5. Ion Content Measurement

The above dried samples with known weight (DW, mg) were used for measurement of Na^+^ and K^+^ ion content. The samples were put into 15-mL Falcon tubes, and 10 mL of 0.1 N hydrochloric acid solution was added. After sample ion solubilization at room temperature overnight, 2 mL of sample solution at 200-fold dilution (10 µL of first sample solution + 2 mL 0,1 N hydrochloric acid solution) was used to measure Na^+^ and K^+^ concentrations (mg/L) by a SpectrAA 220FS atomic absorption spectrometer (Varian, US). The Na^+^ and K^+^ contents (ConcNa and ConcK) were then converted back to the quantity of Na^+^ and K^+^ ions per gram of dry weight (mg/gDW) by the following equations: ConcNa = [Na^+^ measurement (mg/L) × dilution rate (200) × volume of first sample solution (10 mL)]/DW (mg); ConcK = [K^+^ measurement (mg/L) × dilution rate (200) × volume of first sample solution (10 mL)]/DW (mg). The Na^+^/K^+^ ratio (Na_K) was calculated as the proportion of Na^+^ content to K^+^ content.

### 4.3. Statistical Analysis of Phenotypic Data

Statistical analysis of phenotypic data (means, standard deviations, coefficients of variation (CVs), graphs) was carried out in the R software v3.6.2. Analysis of variance (ANOVA) was performed to test the effect of genotype and replication using a linear model of the R function lm(). Broad-sense heritability (H^2^) was used to estimate the genetic variance based on the variance among phenotypic measurements between three replicates of the panel. H^2^ was computed using the following formula: H^2^ = (*F*-value − 1)/*F*-value, where the *F*-value was derived from analysis of ANOVA for the genotype effect [18]. Phenotypic correlations between traits were evaluated by the Pearson method using the corrplot R package. The R function cor.test() was used to test the significance of the correlation coefficients.

### 4.4. Genome-Wide Association Study

The phenotypic data from the salt test and SNP genotypic data on the full panel and the indica and japonica subpanels were separately used to study the marker-trait associations by incorporating a kinship matrix along with population structure. In the Tassel software v.5.0, the structure matrix was determined with 6 axes on the SNP data of the population by running a principal component analysis (PCA). The kinship matrix was built by the pairwise identity-by-state method, to account for relatedness of individuals among 182 accessions. Q-Q and Manhattan plots of the negative log_10_-transformed observed *p*-values for each SNP-trait association were created to visualize the GWAS results. Markers with a *p*-value ≥ 5 × 10^−4^ were declared significant.

The number of QTLs from the detected associations was determined based on linkage disequilibrium (LD) between SNPs surrounding the significant markers. The LD heatmaps were plotted by using the LDheatmap R package, and the genomic regions of QTLs were limited by LD blocks with r^2^ values (squared allele frequency correlation) between SNPs > 0.4. For a low LD block (<50 kb), the interval of QTLs was enlarged by a distance of +/− 50 kb. The qqman package in R software was utilized to highlight the significant markers of strong QTLs in Manhattan plots. The genes in the genomic regions of strong QTLs were scanned in the MSU rice database.

## 5. Conclusions and Future Prospects

Our approach identified different QTLs characterized by the presence of a high number of genes associated with the response to salinity or abiotic stress. Interestingly, these genes are related to hormone transduction pathways or transcriptional modulation of gene expression in response to stress, suggesting that these QTLs act in complementary ways to control the salinity tolerance, which is of major interest for breeding programs. Pyramiding several favorable QTLs in a variety will ensure a better resilience of the plant to salinity stress under different environmental conditions and then a better sustainability of the variety. Therefore, it will be interesting to conduct introgression of the major QTLs identified in this study such as QTL25 in modern varieties cultivated in the Mekong or Red River Delta areas such as Bac Thom 7 and Khang Dan 18. The function of the four *BAK1* genes in QTL25 should be specified by generating single and multiple gene mutations using the CRISPR Cas9 system.

## Figures and Tables

**Table 1:** Phenotypic variation and broad-sense trait heritability for the three panels.

Traits	n	Mean	SD	CV	Replication Effect	Genotype Effect		
					*p*-Value	*p*-Value	*F*-Value	H^2^
Full panel								
WC	182	59.98	10.49	17.49	<0.001	<0.001	3.500	0.71
Score	182	4.41	1.16	26.30	<0.001	<0.001	2.576	0.61
Chl_total	182	1.4	0.65	46.43	<0.001	<0.001	1.924	0.48
Chla	182	0.82	0.35	42.68	<0.001	<0.001	1.902	0.47
Chlb	182	0.52	0.28	53.85	<0.001	<0.001	1.675	0.40
Chla_b	182	1.75	0.99	56.57	0.0560	0.3127	1.063	0.06
ConcK	182	43.39	7.61	17.54	<0.001	<0.001	4.226	0.76
ConcNa	182	18.63	11.97	64.25	<0.001	<0.001	3.470	0.71
Na_K	182	0.42	0.27	64.29	<0.001	<0.001	3.342	0.70
Indica subpanel								
WC	112	58.99	10.74	18.21	<0.001	<0.001	3.570	0.72
Score	112	4.51	1.1	24.39	<0.001	<0.001	2.644	0.62
Chl_total	112	1.39	0.72	51.80	<0.001	<0.001	1.849	0.46
Chla	112	0.82	0.39	47.56	<0.001	<0.001	2.105	0.53
Chlb	112	0.51	0.3	58.82	<0.001	<0.001	1.859	0.46
Chla_b	112	1.82	1.22	67.03	0.0413	0.3759	1.051	0.05
ConcK	112	45.86	7.04	15.35	<0.001	<0.001	2.855	0.65
ConcNa	112	19.48	11.63	59.70	<0.001	<0.001	3.414	0.71
Na_K	112	0.42	0.25	59.52	<0.001	<0.001	2.992	0.67
Japonica subpanel								
WC	64	61.7	9.75	15.80	<0.001	<0.001	2.858	0.65
Score	64	4.26	1.23	28.87	0.9317	<0.001	2.525	0.60
Chl_total	64	1.38	0.52	37.68	<0.001	<0.001	2.292	0.56
Chla	64	0.81	0.29	35.80	0.0026	0.0171	1.580	0.37
Chlb	64	0.52	0.24	46.15	<0.001	0.0414	1.455	0.31
Chla_b	64	1.65	0.43	26.06	0.0072	0.4666	1.014	0.01
ConcK	64	39.38	6.8	17.27	<0.001	<0.001	2.698	0.63
ConcNa	64	17.61	12.41	70.47	<0.001	<0.001	3.056	0.67
Na_K	64	0.44	0.3	68.18	<0.001	<0.001	2.879	0.65

n: number of accessions; Rep: replication; CV: coefficient of variations; H^2^: broad-sense heritability; WC: leaf water content; Score: score of visual salt injury; Chl_total: total chlorophyll content in leaves; Chla: chlorophyll a content in leaves; Chlb: chlorophyll b content in leaves; Chla_b: chlorophyll a to chlorophyll b ratio in leaves; ConcK: K^+^ concentration in leaves; ConcNa: Na^+^ concentration in leaves; Na_K: ratio of Na^+^/K^+^ in leaves.

**Table 2:** Correlation coefficients between traits in the three panels (below the diagonal). Probabilities above the diagonal.

Traits	Panels	WC	Score	Chl_Total	Chla	Chlb	Chla_b	ConcK	ConcNa	Na_K
WC	F	1	<0.001	<0.001	<0.001	<0.001	0.608	<0.001	<0.001	<0.001
WC	I	1	<0.001	<0.001	<0.001	<0.001	0.709	<0.001	<0.001	<0.001
WC	J	1	<0.001	<0.001	0.004	0.012	0.469	<0.001	<0.001	<0.001
Score	F	−0.70	1	<0.001	<0.001	0.004	0.896	<0.001	<0.001	<0.001
Score	I	−0.71	1	<0.001	<0.001	0.004	0.803	<0.001	<0.001	<0.001
Score	J	−0.66	1	0.062	0.093	0.102	0.611	0.033	<0.001	<0.001
Chl_total	F	−0.34	0.17	1	<0.001	<0.001	<0.001	0.065	<0.001	<0.001
Chl_total	I	−0.44	0.24	1	<0.001	<0.001	<0.001	0.027	<0.001	<0.001
Chl_total	J	−0.26	0.13	1	<0.001	<0.001	<0.001	0.507	0.165	0.315
Chla	F	−0.31	0.18	0.82	1	<0.001	0.278	0.231	0.001	0.002
Chla	I	−0.39	0.24	0.85	1	<0.001	0.704	0.042	<0.001	<0.001
Chla	J	−0.21	0.12	0.78	1	<0.001	0.180	0.941	0.664	0.793
Chlb	F	−0.24	0.12	0.75	0.76	1	<0.001	0.845	0.007	0.008
Chlb	I	−0.29	0.16	0.73	0.72	1	<0.001	0.192	<0.001	<0.001
Chlb	J	−0.18	0.12	0.78	0.81	1	<0.001	0.865	0.556	0.739
Chla_b	F	0.02	0.01	−0.27	−0.05	−0.61	1	0.763	0.525	0.591
Chla_b	I	0.02	0.01	−0.24	−0.02	−0.62	1	0.835	0.586	0.638
Chla_b	J	0.05	-0.04	−0.32	−0.10	−0.62	1	0.438	0.611	0.817
ConcK	F	−0.41	0.32	0.08	0.05	0.01	0.01	1	<0.001	<0.001
ConcK	I	−0.43	0.39	0.12	0.11	0.07	0.01	1	<0.001	<0.001
ConcK	J	−0.33	0.15	0.05	0.01	0.01	−0.06	1	<0.001	0.005
ConcNa	F	−0.84	0.63	0.16	0.14	0.11	−0.03	0.42	1	<0.001
ConcNa	I	−0.83	0.63	0.25	0.24	0.19	−0.03	0.41	1	<0.001
ConcNa	J	−0.84	0.59	0.10	0.03	0.04	−0.04	0.41	1	<0.001
Na_K	F	−0.79	0.59	0.15	0.13	0.11	−0.02	0.20	0.96	1
Na_K	I	−0.79	0.61	0.23	0.22	0.18	−0.03	0.23	0.97	1
Na_K	J	−0.81	0.59	0.07	0.02	0.02	−0.02	0.20	0.97	1

F: full panel; I: indica subpanel; J: japonica subpanel; WC: leaf water content; Score: score of visual salt injury; Chl_total: total chlorophyll content in leaves; Chla: chlorophyll a content in leaves; Chlb: chlorophyll b content in leaves; Chla_b: chlorophyll a to chlorophyll b ratio in leaves; ConcK: K^+^ concentration in leaves; ConcNa: Na^+^ concentration in leaves; Na_K: ratio of Na^+^/K^+^ in leaves.

## Data Availability

The GBS genotyping dataset supporting the results of this study has been deposited as a downloadable Excel file in TropGeneDB: http://tropgenedb.cirad.fr/tropgene/JSP/interface.jsp?module=RICE (accessed on 24 May 2021) tab “studies”, study type “genotype”, study “Vietnamese panel-GBS data”. The seeds of the accessions are available in the National Key Laboratory for Plant Cell Biotechnology of Agricultural Genetics Institute, Hanoi, Vietnam.

## Supplementary Materials

The following are available online at https://www.mdpi.com/article/10.3390/plants10061088/s1, Figure S1: Correlation plots in the full panel and the indica and japonica subpanels, Figure S2: Manhattan plots and Q-Q plots for GWAS of salinity tolerance-related traits in the indica panel. In the Manhattan plots, significant SNPs are highlighted in red, Table S1: GWAS associations and significant SNPs at *p* ≤ 1 × 10^−3^ in the full panel and the indica subpanel, Table S2: Colocalizations of the QTLs identified in this study with previous reports, Table S3: Colocalizations of the QTLs detected in this and previous studies using the same rice panel and genotyping data, Table S4: List of the 183 rice accessions used in the experiment.
