# Supplementary figures and images for: Genome-Wide Association Mapping of Salinity Tolerance at the Seedling Stage in a Panel of Vietnamese Landraces Reveals New Valuable QTLs for Salinity Stress Tolerance Breeding in Rice
Source: Plants (Basel). 2021 May 28;10(6):1088. doi: 10.3390/plants10061088 (PMC8228224; doi:10.3390/plants10061088)

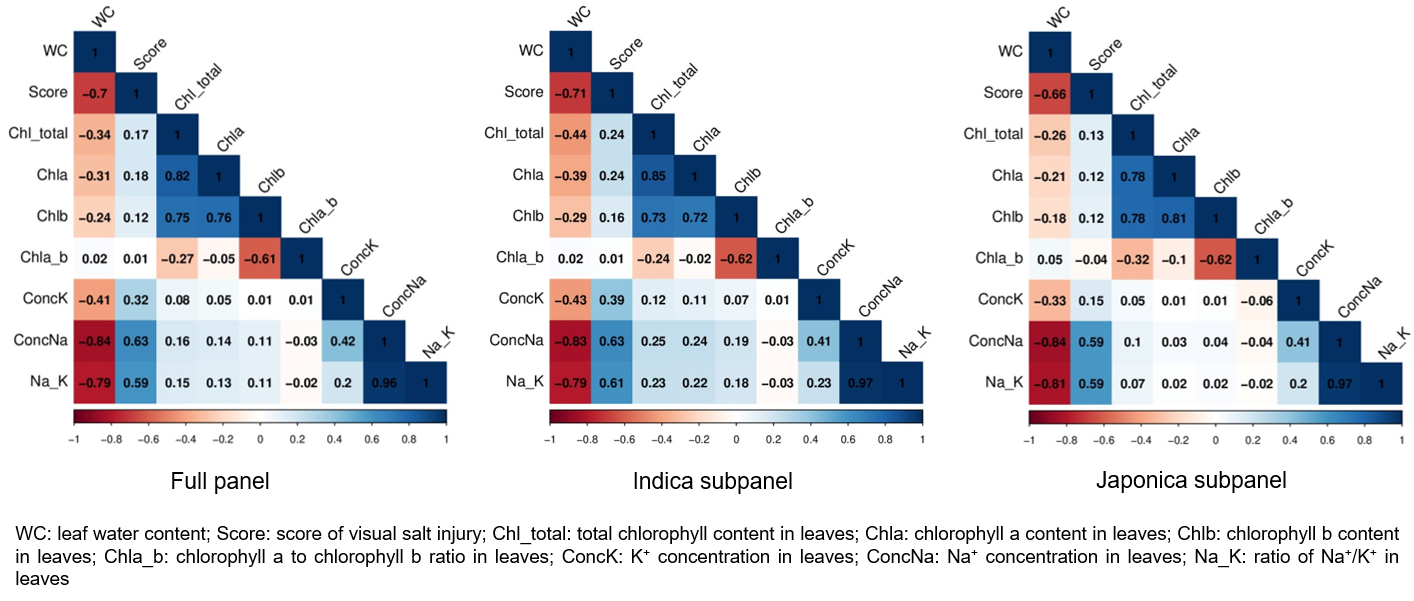

Supplement: Supplementary file 1 [file plants-10-01088-s001.zip › plants-1203594-supplementary/Figure S1.tif]

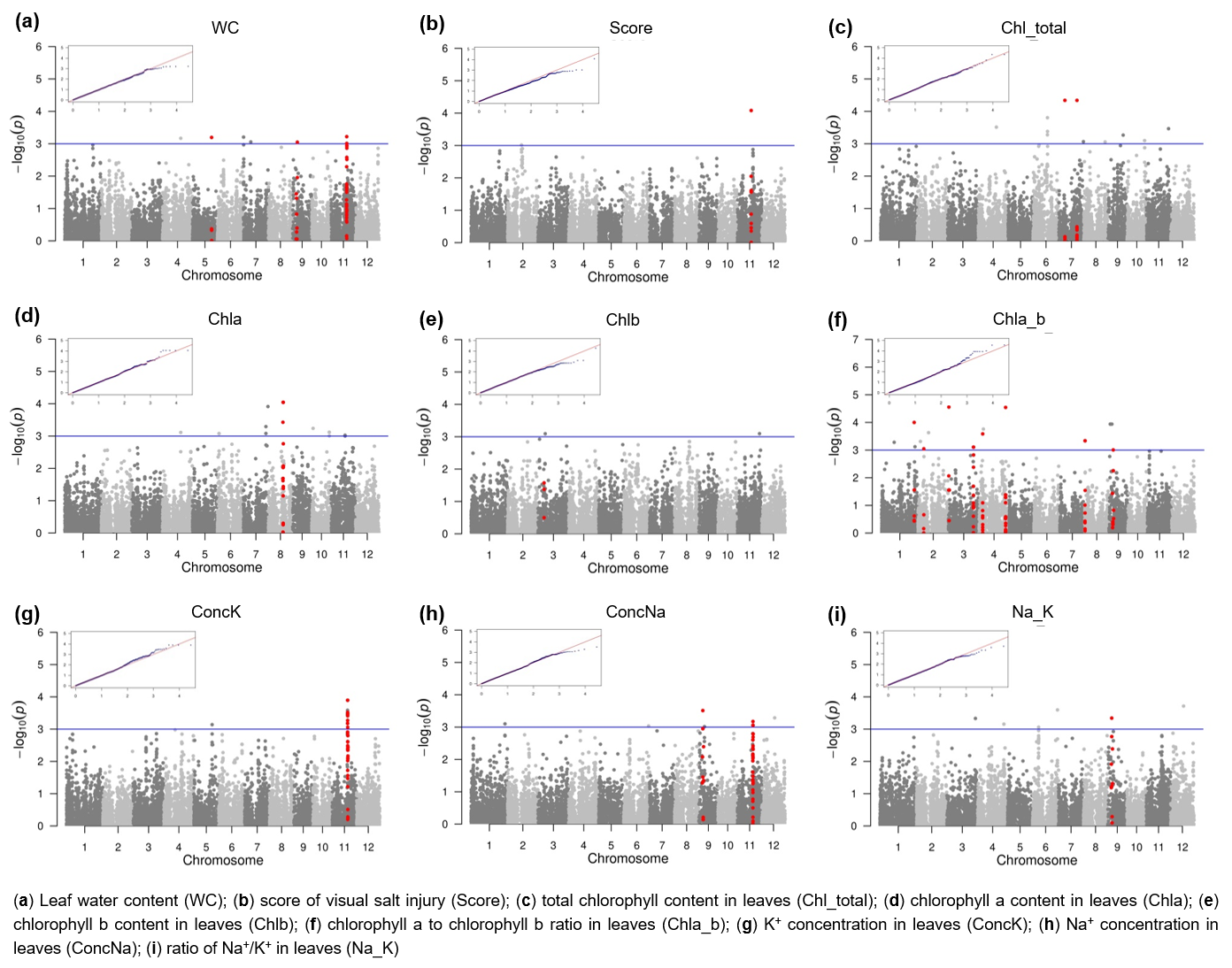

Supplement: Supplementary file 1 [file plants-10-01088-s001.zip › plants-1203594-supplementary/Figure S2.tif]
